# Students engagement using polls in virtual sessions of physiology, pathology, and pharmacology at King Saud bin Abdulaziz University for Health Sciences during COVID-19 pandemic: a cross-sectional study

**DOI:** 10.1186/s12909-023-04253-w

**Published:** 2023-04-21

**Authors:** Mona Abubakr Bawazeer, Saima Aamir, Fatmah Othman, Reem Alkahtani

**Affiliations:** 1 College of Medicine, King Saud Bin Abdulaziz University for Health Sciences (KSAU-HS), Riyadh, Saudi Arabia; 2 King Abdullah International Medical Research Centre (KAIMRC), Ministry of National Guard Health Affairs (MNGHA), Riyadh, Saudi Arabia; 3 College of Public Health and Health Informatics, King Saud Bin Abdulaziz University for Health Sciences (KSAU-HS), Riyadh, Saudi Arabia

**Keywords:** Poll, Virtual, Engagement, Understanding, Medical students

## Abstract

**Background:**

Active involvement of students in class using technology is associated with effective learning and understanding. This work intended to analyze the impact of interactive teaching on medical students’ engagement, learning, performance, understanding and attendance in virtual classes of physiology, pathology, and pharmacology during COVID-19 pandemic.

**Methods:**

A descriptive cross-sectional study was carried out at college of medicine at King Saud bin Abdulaziz University for Health Sciences (KSAU-HS) in Riyadh during January-April 2022. Third- and fourth-year medical students filled a self-reported questionnaire that assessed students’ engagement, understanding, performance, and attendance during the sessions of three courses within the curriculum. The Chi-square test or Fisher’s exact test was used to compare the difference between the survey responses.

**Results:**

A total of 184/234 questionnaires were completed and returned, with an overall response rate of 78.6%. Fifty-five percent of the participants were involved at least more than 5 times in polls during the class. Majority (86.9%), of the students agreed on enjoying participation in polls during the class, and 88.9% recommended the utilization of the polls again. Participation in polls improved understanding and performance of 88%, and 63% of students respectively. In addition, 38% were neutral regarding attendance improvement and spending more time for the class. Around 53% students agreed that polls improved their grades.

**Conclusion:**

In conclusion, this study showed that there is an impact of using interactive polls in virtual classes in medical students at KSAU-HS. It is recommended to continue using polls in all subjects in on-site sessions. This will be a great preface step toward switching the traditional teaching to the interactive teaching using flipped classroom strategy in the future.

## Background

There is a trend of change from teacher-centered learning to student-centered methodology [1]. Classroom interaction is one of the potential areas to focus on, in order to improve the learning environment [2]. Interactive (active) learning is a process, where students are engaged in activities that encourage them to think, reflect, assess their understanding and problem-solving skills [3]. The impact of interactive and traditional teaching on students’ cognitive outcomes was evaluated by Michel et al. They concluded that the interactive teaching approach may influence more positive feedback on students’ learning [4]. Interactive teaching demonstrated significant improvement of students' satisfaction and exam grades [5]. Mains et al. studied impact of inserting three multiple choice questions related to knowledge and comprehension in a recorded lecture on presentation and management of burns, using an electronic system to receive immediate response from students. They observed improvement in students' engagement, understanding and perception [6].

Students can interact with the teachers during the class by asking questions and responding to their queries. But students are usually reluctant and apprehensive of committing mistakes in front of others. Nevertheless, it is not always possible for the tutors to gather responses from all students especially when dealing with substantial number of students in the class. Several studies have demonstrated application of polling and student response systems in social sciences. They are considered to be popular methods for gathering considerable replies in a brief span of time [7]. Students’ responses are usually anonymous and the results can be displayed in graphical form in real time [8]. Students’ concentration and engagement remarkably improved after introduction of gaming software in the classes. They acquired profound interest in the subject, and they were able to exchange information freely with their instructor and classmates [9–13]. Many authors have illustrated an optimistic shift in the traditional classroom setting after introduction of game-based quizzes in medical education as well. This results in improving students’ participation, motivation and allows the instructor to identify the gaps in students' knowledge and comprehension to clarify the misconceptions. Active involvement in class is associated with effective learning and retaining knowledge. Overall, a pleasant, and cheerful atmosphere is witnessed, and the students feel excited that help to boost their confidence and eagerness to learn [14–17].

The SARS-CoV-2 pandemic had a substantial impact on educational institutions globally. This unexpected situation substituted the conventional teaching and learning with an alternative pedagogical approach. Academic institutes in the Kingdom of Saudi Arabia were instructed by the Ministry of Education to deliver the curriculum through remote learning and online classes in March 2020 [18]. This paradigm shift presented challenges for both faculty and students. Faculty members had to align academic activities with use of technology in virtual teaching [19]. Students became distracted and had a reduced ability to concentrate and focus during online teaching. It has been demonstrated that integration of technology and interactive learning activities results in better engagement of students during online learning. Student engagement and interaction is a fundamental component of academic learning [11]. Studies have shown that a Classroom Response System like Kahoot! can be effectively used in current pandemic. It does not only boost attention, interaction and participation during the session but also provides immediate feedback to the students and instructors. This encourages the students to evaluate their knowledge, resulting in enhanced analytical and reasoning skills [20, 21]. Martin-Sómer et al. demonstrated that utilization of Kahoot! during the pandemic facilitated students’ learning and had a favorable impact on understanding and exam results during virtual teaching [22]. Polls are useful not only in evaluating the students’ comprehension during the class but also help in assessing the knowledge gained in the previous session. They can facilitate the educators to identify the most frequent mistakes and address them accordingly. Jamil et al. conducted a study on implementation of Kahoot! -based quizzes in undergraduate medical curriculum. They recognized a distinct change in students’ attitude towards learning and active participation in the class while the instructors fostered critical reasoning by addressing misconceptions and reviewing problematic topics. They found it to be a valuable tool in recognizing and overcoming shortcomings in understanding the course content [17]. Analogous observations have been mentioned by other authors who have established that interactive polls helped to consolidate the concepts resulting in better academic progress, augmented self-esteem, and critical thinking; especially benefitting below average students [16, 23, 24].

Aim of this research was to analyze the impact of interactive teaching in undergraduate medical curriculum of physiology, pathology, and pharmacology in third- and fourth-year medical students in virtual classes during SARS-CoV-2 pandemic at College of Medicine, King Saud bin Abdulaziz University for Health Sciences (KSAU-HS), Riyadh. Students’ engagement, learning, performance, understanding and attendance was analyzed. Scanty research is available to ascertain the usefulness of the interactive polls and quizzes in remote learning during the pandemic. Consequently, it is essential to determine the utilization of student response systems to establish their efficacy in achieving better learning outcomes.

## Method

### Study setting and population

A cross-sectional study was conducted with undergraduate students in the college of medicine, KSAU-HS, from January to April 2022 to analyze the impact of interactive teaching of physiology, pathology, and pharmacology on medical students’ engagement, learning, performance, understanding and attendance in virtual classes during SARS-CoV-2 pandemic. Students at KSAU-HS are enrolled in two streams. Stream I students are accepted directly after high school while students holding a bachelor’s degree are grouped as stream II constituting 10% of the whole batch. The curriculum of the medical program consists of three phases including pre-professional and the professional phase followed by one year of medical internship. Students receive basic science and English courses in the pre-professional phase over a period of two years in College of Science and Health Professions (COSHP). The professional phase within college of medicine is divided into two years each of “pre-clinical phase II” and “clinical phase III”. In Saudi Arabia, the Ministry of Education (MOE) designs colleges with male and female campuses, in which both campuses are equipped equally and the education level is assured to be the same. Therefore, at KSAU-HS there is college of medicine in the male and female campuses. In our study, all the female undergraduate students in the preclinical phase II were included. A convenient sampling method was used to choose the students from the program. The estimated sample size was calculated as 148 students using a total population size of 234, 5% margin of error, and 95% confidence level with the help of Raosoft sample calculator (http://www.raosoft.com/samplesize.html). The study was approved by the Institutional Review Board (IRB), Ministry of National Guard Health Affairs (IRB approval No. IRB/0102/22). Informed consent was obtained from each student prior to completing the questionnaire.

### Study instrument

**Fig. 1:**
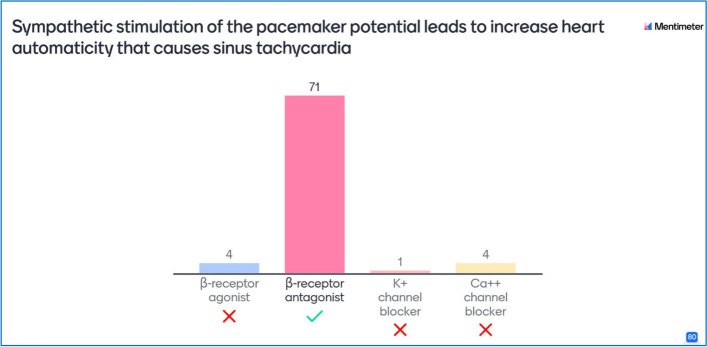
Screenshot showing a multiple-choice question with students’ responses using Mentimeter

**Fig. 2:**
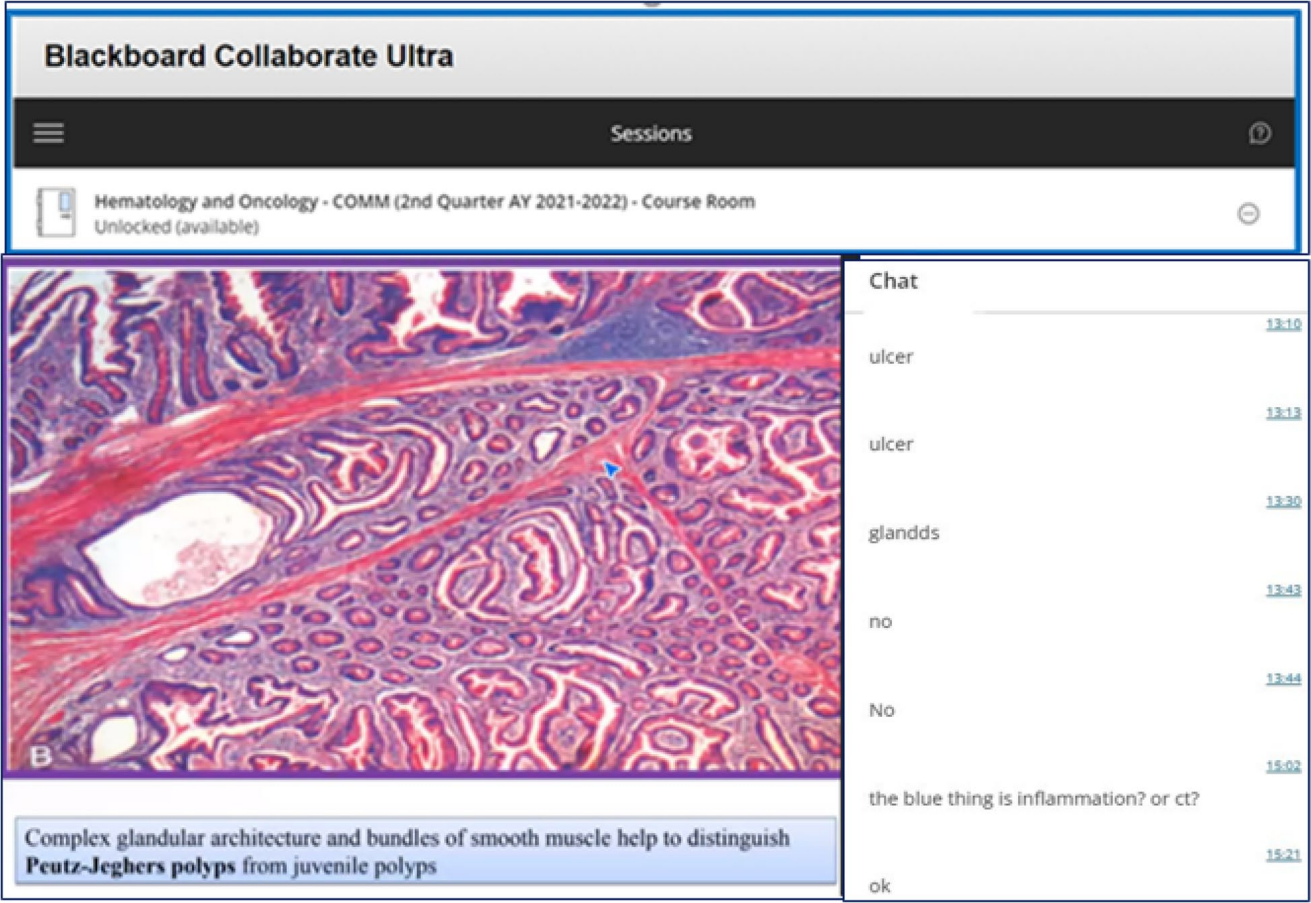
Screenshot showing students’ interaction with instructor through chat in Black Board Collaborate Ultra during a virtual session in pandemic

The faculty members of physiology, pathology, and pharmacology utilized different classroom-response systems such as blackboard polls and chat, Kahoot! or Mentimeter (Figs. 1 and 2) for interaction with students at the beginning, throughout, and the end of the virtual sessions for two semesters. Kahoot! and Mentimeter are online audience response systems based on game-based quizzes and multiple choice questions to facilitate students’ engagement and learning. Blackboard is a learning management system with inbuilt tools including delivery of virtual sessions and polls for interaction with the students. These basic medical science courses are offered in pre-clinical phase (phase II) in various block modules for two years. The self-administered validated questionnaires were distributed to the participants after the second semester at end of the courses to assess the students' engagement in the virtual sessions using polls. A validated questionnaire adapted from the published work was used [25]. Written approval was taken from the corresponding author. The questionnaire consisted of 11 questions; the first 5 questions assessed participants’ engagement in interactive classes. The second 5 questions evaluated students’ performance as well as understanding. Finally, the last question assessed whether the interaction in sessions affected students’ attendance. Hard copies of the questionnaire were distributed to the students.

### Statistical analysis

Descriptive statistics were used to describe the participants’ responses for each item in the questionnaire. The Chi-square test or Fisher’s exact test was used to compare the difference between the survey responses (agree, neutral, and disagree) and the study year of the students. Initially the questionnaire had a five-point Likert scale (strongly agree, agree, neutral, disagree, strongly disagree) then it was re-categorized into three categories after receiving the responses: “Agree” (including strongly agree and agree), “Disagree” (strongly disagree and disagree), and “Neutral” category. Results were considered to be statistically significant if the *p*-value was < 0.05. All analyses were done using STATA version 12 (StataCorp. 2011. Stata Statistical Software: Release 12. College Station, TX: StataCorp LP).

## Results

A total of 234 questionnaires were distributed to students in phase II (third- and fourth- year) who were taking classes of physiology, pathology, and pharmacology, 184 of which were completed and returned, with an overall response rate of 78.6%. A total of 184 students participated in the study. Third-year students constituted 53.8% of the sample, where 88.5% of the participants belonged to Stream I (Table 1). Majority of the respondents (55%) indicated that they participated at least more than 5 times in polls during the virtual classes.

**Table 1:** Basic description of participants’ characteristics

Characteristics	Number (%) *N* = 184
**Year of study**	
Third Year	99 (53.8)
Fourth Year	85 (46.2)
**Stream**	
Stream I	163 (88.5)
Stream II	21 (11.5)
**Number of participation in the polls**	
1–2	27 (14.6)
3–4	54 (29.3)
> 5	103 (55.9)

Table 2 displays breakdown of the students’ responses about engagement in interactive classes. Majority of the students agreed on their enjoyment in participating in polls during the class (86.9%), and they recommended to utilize the polls again (88.9%). Comparing to third-year students, fourth-year students were less likely to agree, *p*-value < 0.001).

**Table 2:** Response percentages of items related to engagement in interactive classes

Item	Third Year *N* = 99(%)	Fourth Year *N* = 85(%)	Total *N* = 184(%)	*p*-value
**Enjoyed participating in polls**				
Agree	86(86.8)	74(87.1)	160(86.9)	0.897
Neutral	11(11.1)	10(11.7)	21(11.4)	
Disagree	2(2.1)	1(1.1)	3(1.6)	
**Recommend polls again**				
Agree	88(88.9)	74(87.1)	162(88.1)	0.591
Neutral	10(10.1)	8(9.4)	18(9.7)	
Disagree	1(1.1)	3(3.5)	4(2.1)	
**Recommend polls to be used in other subjects**				
Agree	84(84.8)	44(51.7)	128(69.5)	**< 0.001**
Neutral	13(13.1)	28(32.9)	41(22.2)	
Disagree	2(2.1)	13(15.2)	15(8.1)	
**Polls improved interaction with other students**				
Agree	89(89.9)	74(87.1)	163(88.5)	0.815
Neutral	9(9.1)	10(11.7)	19(10.3)	
Disagree	1(1.1)	1(1.1)	2(1.1)	
**Polls improved interaction with instructor**				
Agree	50(50.5)	43(50.5)	93(50.5)	0.247
Neutral	42(42.4)	30(35.2)	72(39.1)	
Disagree	7(7.1)	12(14.1)	19(10.3)	

Usefulness of polls in enhancing students’ performance and understanding is illustrated in Table 3. Eighty-eight percent of the students agreed that participation in polls improved their understanding of subjects, where 63% of the students agreed that polls improved performance. Ninety-one percent of the students agreed that feedback from instructors about solutions of polls helped in understanding multiple choice questions in the exams. About 38% participants were neutral regarding role of polls in improving class attendance, similarly 38% were neutral about spending more time in preparation for the class involving polls. Compared to third-year students, fourth-year students were in more disagreement as regards to allocating more time for preparedness for the virtual sessions utilizing polls (49% for the fourth year compared to 22% for the third year, *p*-value < 0.001). Around 53% students agreed that polls improved their grades. While 18% of fourth-year students disagreed that polls improved the grades compared to 4.1% of third-year students, *p*-value 0.004.

**Table 3:** Response percentages of items related to students’ performance and understanding

Item	Third year *N* = 99(%)	Fourth Year *N* = 85(%)	Total *N* = 184(%)	*p*-value
**Polls participation improved understanding of subjects**				
Agree	89(89.9)	74(87.1)	163(88.5)	0.815
Neutral	9(9.1)	10(11.7)	19(10.3)	
Disagree	1(1.1)	1(1.2)	2(1.1)	
**Polls improved performance**				
Agree	62(62.6)	54(63.5)	116(63.1)	0.268
Neutral	33(33.4)	23(27.1)	56(30.4)	
Disagree	4(4.1)	8(9.5)	12(6.5)	
**Polls improve grades**				
Agree	54(54.5)	43(50.5)	97(52.7)	**0.004**
Neutral	41(41.2)	26(30.6)	67(36.4)	
Disagree	4(4.1)	16(18.8)	20(10.8)	
**Spending more time for classes using polls**				
Agree	29(29.3)	21(24.7)	50(27.1)	**< 0.001**
Neutral	48(48.4)	22(25.8)	70(38.1)	
Disagree	22(22.2)	42(49.4)	64(34.7)	
**Feedback from instructors about solution of polls helped in understanding MCQ**				
Agree	93(93.9)	75(88.3)	168(91.3)	0.359
Neutral	5(5.1)	7(8.2)	12(6.5)	
Disagree	1(1.1)	3(3.5)	4(2.2)	
**Attendance improved in the subjects using polls**				
Agree	48(48.4)	41(48.2)	89(48.4)	**0.011**
Neutral	44(44.4)	26(30.5)	70(38.1)	
Disagree	7(7.1)	18(21.1)	25(13.5)	

## Discussion

Utilization of online polling platforms is considered to be an invaluable and versatile means for acquisition of knowledge in medical education [21, 26]. The adoption of emerging gamified learning modalities has been shown to augment conventional instructional strategies and reinforce effective teaching in physiology [27], pharmacology [28], histology [29], pathology of benign and malignant tumors [30, 31], oral pathology [15], immunology [32], and intramuscular injection courses [33]. Education trends combined with technology can help us incorporate rich interactive experiences in our teaching. This study was designed to document the impact of interactive teaching utilized in physiology, pathology, and pharmacology virtual classes during SARS-CoV-2 pandemic among the third- and fourth- year medical students at KSAU-HS.

Majority of respondents in our research (86.9%) acknowledged that they experienced pleasure in polls participation during online classes. We integrated polling activities in virtual sessions during the pandemic and as educators we were able to transform monotonous teaching environment into a pleasurable one filled with fun and amusement, simultaneously increasing the engagement and participation rate among students. Students’ perception and satisfaction can be depicted from students’ comments received in chat section of Blackboard during an online session delivered in the pandemic. Some of the students commented that “this was one of the best and fun filled session in the block”, others appreciated the interaction. Ismail et al. reported that utilization of Kahoot! in sessions was found to be delightful for the students [16]. Likewise, Ali et al. also established that introduction of interactive polls in oral pathology lectures through user friendly tools was gratifying for the students [15]. Similarly a study conducted by Ofori et al. on application of polling technology in PharmD course demonstrated that around 84% survey respondents believed this interaction to be enjoyable and exciting [34].

According to our research, 88% of returned questionnaires indicated that incorporation of interactive activities in virtual sessions accelerated understanding of learning material resulting in enriched quality of learning during SARS-CoV-2 pandemic. Polls is an amazing digital tool that allows us to improve communication, enhance memorization, and provide instant feedback. This in turn, increased students’ participation, satisfaction, customized learning, and class motivation. Students participating in quiz-based games in pathology, performed substantially better in the final assessments and they were considered as an admirable means by the students for reinforcing course content and effective discussion [30, 31]. Ali et al. recognized that utilization of innovative interactive techniques in oral pathology encouraged students in easy identification of pathological lesions [15]. Ofori et al. analyzed impact of Kahoot in Pharm-D course and they discovered that 71% students had improved perception of the subject which led to enhanced knowledge acquisition and learning outcomes achievement [34]. Correspondingly, Sumanasekeraa et al. acknowledged that most of the students recommended to include interactive strategies like quizzes in teaching pharmacology courses rather than conventional instructional techniques [28]. Likewise, Phelps et al. explored the use of interactive quizzes in virtual teaching of Physiology course in an Australian university. They determined that active involvement of students in these activities empowered them to participate more in the classroom resulting in better engagement, and attention span [35].

**Fig. 3:**
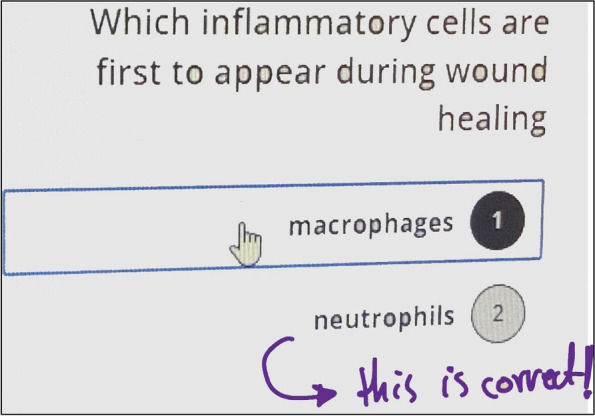
Screenshot from Black Board Collaborate Ultra showing student’s response to polls after instructor’s feedback

Our study revealed that most of the participants agreed that the interpretation of questions in polls and the constructive feedback by faculty members amplified the ability to analyze and understand multiple choice questions in summative assessments.Application of polls in basic medical sciences has been proved to be a joyful teaching strategy, which boosted students’ engagement and motivation. This reduced the sense of isolation and remoteness during the corona virus outbreak. This also assisted in providing instant feedback which is a powerful tool for learning and correcting misconceptions as revealed in Fig. 3. Nayak et al. demonstrated that interpretation of interactive questions by the instructors during radiology class discussion boosted learning and increased eagerness to learn [36]. Implementation of quiz-based games in course of oral pathology and intramuscular injection stimulated the students to develop enhanced skills and competencies resulting in significantly improved performance in the examination [15, 33]. These quick quizzes via polls helped to maintain the students’ attention in class and assisted the instructors to make sure that students have understood the lesson delivered and increased their participation during classes.

Addition of polls during basic medical sciences sessions helped us to improve curriculum delivery via interactive and instant feedback. Regardless of the teaching style, interactivity and reflection allowed us to break the rigid structure of classic medical science teaching. About 88.9% of medical students in our study expressed that they would like to have polls for effective communication with the instructors in upcoming sessions. Most of the students in third year proposed to adopt interactive questions in other subjects as well. Freitas et al. concluded that 90% of students supported inclusion of Kahoot! in anatomy course to reinforce understanding of the subject [23]. Correspondingly, Kelleny et al. studied effect of gamification-based technology in teaching during pandemic. They revealed that almost 98.5% students preferred to employ this form of interaction in all teaching and practical sessions [20]. Furthermore, a significant number of students advocated application of Kahoot! in teaching pathology in Helwan University, Egypt [31].

Communication is the core of learning, it is the process of understanding and sharing information among the students and faculty. Sometimes, it can be hard to communicate effectively with students, specifically when we have to adapt to a large number of students in class or during distance learning. Since polls uses both verbal and visual communication tools, so they tend to improve interactivity. Majority of the students in our study population had an agreement on the fact that utilization of polls in virtual sessions improved interaction with other students (88.5%) and 50.5% believed that there was better communication with the instructor. Correspondingly, a study was organized in Aga Khan university Pakistan to evaluate the impact of Kahoot! quizzes in teaching of undergraduate medical students. The results revealed that 80% students affirmed that Kahoot! quizzes allowed them to make comparisons of their knowledge acquisition to that of their classmates in an anonymous state without anxiety of being judged [17]. Introduction of game-based technology in oral pathology and histology courses resulted in better students’ engagement with their classmates and teachers leading to an optimistic approach towards the subject [15, 29].

Most of students in our survey didn’t believe that they spent more time in preparation for the class involving polls. This finding is in comparison with Jamil et al. who also demonstrated that the majority of student in their study stated that they came unprepared for the sessions [17]. In contrast, Ali et al. reported that approximately 97.3% of students studied the lecture content before attending the sessions of oral pathology [15]. The answers for spending more time for classes using poll were dispersed. This could be due to confused statement. It might be understood as spending more time for studying, or for class preparation. For future study, this statement is suggested to be modified to “spending more time for classes preparation prior to class” to avoid the misunderstanding. In addition, 48% of participants agreed that polls improved their class attendance. The study was conducted during virtual classes, which may affect students’ attendance as well. Although, a study done at Harvard school of dental medicine stated that there was no change of the students’ perceptions of attendance with the virtual lectures [37]. However, technical challenges may alter attendance in virtual sessions [38].

An optional and general feedback was received from 92 participants at the end of the questionnaire. Most of the students stated that introduction of polls in classes was helpful, pleasurable, and improved understanding, engagement, motivation, and attention especially in virtual sessions during the pandemic. Student found the class engagement during polling activity to be very appealing and an effective learning style as they have mentioned in their comments that they felt more comfortable. Interestingly, one participant has mentioned that polls increased her confidence. When she found her answer was correct, she was motivated to study further in order to participate more in the next class. In fact, there is high correlation between confidence and improvement of students’ performance [39]. This interactive and visual communication could attribute to the enhancement of memory over a longer period of time. Another participant has mentioned that polls were very effective in understanding the materials but not much helpful for the exam.

One of the limitation in our study is that it lacked the impact of active teaching on students’ grades. The curriculum of medicine program at KSAU-HS is built on problem-based learning (PBL). In this teaching method, students are assessed for all subjects integrated using formative and summative assessments [40]. Therefore, it is challenging to evaluate the grades for the three disciplines (physiology, pathology, and pharmacology) separately. Another limitation is that the participants were only females in phase II (third- and fourth- year). This is because the faculties who participated in this study teach at the female campus in KSAU-HS and specialized in basic medical sciences. Basic medical sciences are covered in phase II years as per the college curriculum. However, we are looking to involve both campuses in future studies as well as classes that are taught in phase III years.

## Conclusion

In conclusion, this study showed that there is an impact of using interactive polls in virtual classes of physiology, pathology, and pharmacology during SARS-CoV-2 pandemic. The impact was evaluated on students’ engagement, understanding, performance, and attendance of medical students at KSAU-HS. Authors plan the continuous use of polls in their on-campus classes after the pandemic and encourage other faculty members at college of medicine in KSAU-HS to apply this technique. This will be a great start and a preface step toward switching the traditional teaching to the interactive teaching using flipped classroom strategy in the future.

## Data Availability

The datasets analyzed during the current study are available from the corresponding author on reasonable request.
